# Perceptions of Healthcare Quality in Duchenne Muscular Dystrophy: A Patient Experience Exploratory Study

**DOI:** 10.3390/healthcare13040412

**Published:** 2025-02-14

**Authors:** Miriam Pavelth Casillas-Ávila, Ileana Chavez-Maisterra, Benjamín Gómez-Díaz, Vanessa Ivonne Orellana Villazon, Rosa Elena Escobar-Cedillo, Alexandra Berenice Luna-Angulo, Edgar Oswaldo Zamora González, Norma Alejandra Vázquez-Cárdenas, Marlen Escotto-Ramírez, Georgina Martínez-Gómez, Luz Berenice López-Hernández

**Affiliations:** 1 Hospital Civil “Dr. Antonio González Guevara”, Tepic 63169, Mexicoedgar.zamora8148@academicos.udg.mx (E.O.Z.G.); 2 Departamento Académico de Ciclo de Vida, Universidad Autónoma de Guadalajara, Av Patria 1201, Zapopan 45129, Mexico; ileana.chavez@edu.uag.mx (I.C.-M.); alejandra.vazquez@edu.uag.mx (N.A.V.-C.); marlen.escotto@edu.uag.mx (M.E.-R.); georgina.martinez@edu.uag.mx (G.M.-G.); 3 Instituto Nacional de Rehabilitación Luis Guillermo Ibarra Ibarra, México City 14389, Mexico; bgodiaz@gmail.com (B.G.-D.); rescobarmex@gmail.com (R.E.E.-C.); lunangulo@gmail.com (A.B.L.-A.); 4 James M. Anderson Center for Health Systems Excellence, Cincinnati Children’s Hospital Medical Center, Cincinnati, OH 45229, USA; vanessa.orellanavillazon@cchmc.org; 5 Departamento de Bienestar y Desarrollo Sustentable, Centro Universitario del Norte de la Universidad, Guadalajara 44214, Mexico

**Keywords:** healthcare quality, patient experience, perceptions

## Abstract

Background: Duchenne muscular dystrophy (DMD) is a genetic disorder characterized by progressive muscle weakness, a decline in quality of life, and premature mortality. This study aims to evaluate the perceived quality of healthcare and the experience of DMD patients and their caregivers in Mexico, comparing governmental and non-profit healthcare institutions using a newly designed assessment instrument. Methods: In a cross-sectional study, data were gathered from 91 participants through an online self-administered questionnaire informed by the Donabedian model and six dimensions of healthcare quality. Results: Analysis revealed two key mediating factors: perceived quality of healthcare and patient experience. The Mann–Whitney U test showed that non-profit organizations were perceived to provide superior quality care in both aspects (*p* < 0.05), notably regarding healthcare professionals’ preparedness and other domains of quality. However, the patient-centered care domain indicated that the importance of quality domains may vary according to cultural and social contexts. In Mexico, the humanistic approach of healthcare professionals appeared to compensate for shortcomings in timely diagnosis and other quality domains. This emphasizes the necessity for enhanced patient education and healthcare evaluation, and it highlights that patient satisfaction does not always correlate with high-quality healthcare. The developed instrument can further aid in understanding the experiences of DMD patients across different countries and cultures.

## 1. Introduction

Duchenne muscular dystrophy (DMD) is a rare, x-linked disorder that affects males, while females typically remain asymptomatic carriers. DMD is characterized by progressive muscle weakness, significant disability, and premature death [1]. Providing high-quality healthcare for patients and families affected by DMD requires a multidisciplinary approach. This includes timely and accurate diagnosis; opportune initiation of steroid therapy [1,2,3]; and long-term management, including physical therapy, cardiology, and respiratory care [2], as well as psycho-social support for patients [3]. Female carriers also require genetic testing, counseling, and psycho-social support due to the potential implications for family planning and emotional burden [4]. Patients with DMD and their families are more vulnerable to emotional distress and financial burden than families of healthy children because they have to deal with a disabling condition that affects their psychosocial sphere and significantly modifies their life quality and expectancy [5]. DMD was associated with a nearly tenfold increase in healthcare costs [6]. This increase was particularly pronounced in individuals with advanced disease stages, characterized by non-ambulation and ventilator dependence. These challenges are pronounced in low- and middle-income countries like Mexico, where systemic healthcare barriers, such as limited resources, delays in diagnosis, and inequitable access to care, exacerbate the difficulties of managing DMD [7].

While several reports highlight delays in diagnosis [8] and document the lived experiences of DMD patients [9], limited research explores information on how patients and families perceive the quality of healthcare they receive. Recently, there has been a growing interest in understanding the patient experience (PE) as a component of healthcare quality, given its link to better health outcomes and its role in identifying gaps in care delivery [10,11,12].

### 1.1. The Patient-Experience Concept

PE refers to the broad spectrum of interactions that patients have with the healthcare system, including encounters with physicians, nurses, and other staff in a variety of settings, such as hospitals, physician offices, and healthcare facilities [12,13]. PE encompasses several dimensions, such as communication with healthcare professionals, access to resources and services, and convenience and overall quality of care received [13]. Importantly, PE extends beyond the dimension of patient-centered care; it captures the overall impact of healthcare on patients, encompassing their perceptions and the various factors involved. While a study on DMD provided a roadmap for patient-centered DMD drug development [14], no studies have focused on the global perception of the overall healthcare received by patients and families with DMD.

Healthcare quality (HQ) focuses on the degree to which services improve the likelihood of achieving optimal health outcomes for individuals and populations. HQ relies on evidence-based practices and encompasses all stages of care, including health promotion, prevention, treatment, rehabilitation, and palliative care. The overarching goal of HQ is to maximize patient benefits and minimize potential risks [15]. Since they share the goal of improving patient outcomes, PE and HQ are intrinsically linked concepts within health systems. Both are essential to assess and improve the effectiveness of healthcare services and ensure that care is patient-centered and outcome-oriented. Understanding the experiences of families and patients with DMD would contribute to healthcare-quality improvement.

### 1.2. The Avedis Donabedian Model and the Six Domains of Healthcare Quality

**Figure 1:**
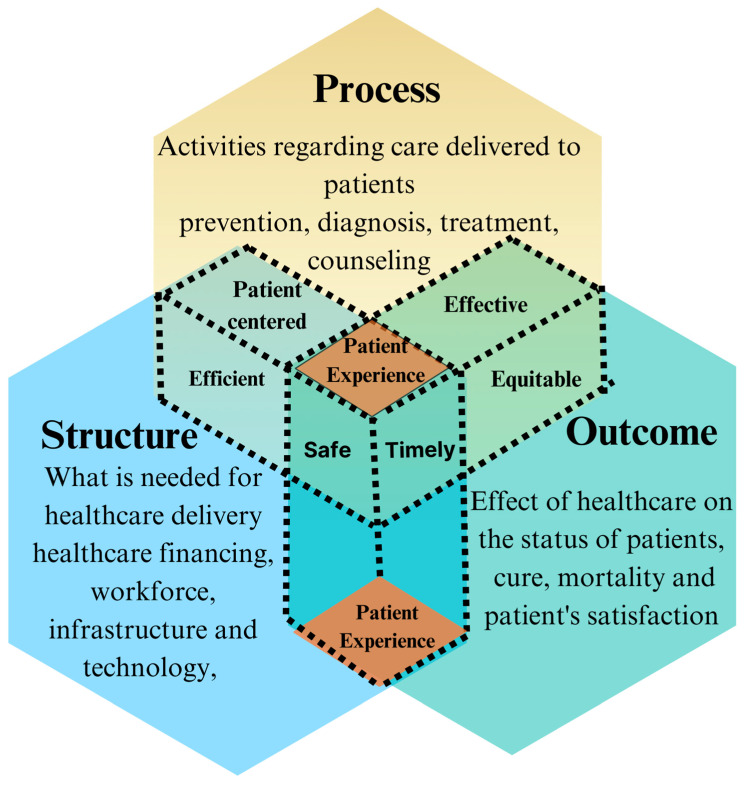
The SPO model of Avedis Donabedian and the Six Domains of Quality Healthcare in the context of patient experience.

The Avedis Donabedian Model provides a widely recognized framework for understanding and assessing healthcare quality, focusing on three core components: structure, process, and outcome (SPO) [16]. Structure refers to the resources and characteristics of the healthcare system, including the availability and adequacy of facilities, equipment, organizational culture, and the qualifications of medical staff. Process encompasses the actions taken to deliver care and implies the interaction between patients and healthcare professionals, such as patient communication, consultations, surgeries, and discharges. Outcome evaluates the results of healthcare services on patient status, including indicators like satisfaction, survival rates, recovery, and mortality. Improvements in structural elements such as modern medical equipment, adequate staffing, and clear procedural guidelines can enhance clinical processes, ultimately leading to better patient outcomes and experiences [15,17]. The outcome is significantly influenced by both the structure and the process; thus, our study places a central focus on processes as a pivotal component of the PE (Figure 1).

In addition to the SPO model, the six domains of healthcare quality provide another comprehensive framework for evaluating care [18]. These domains include safety (minimizing harm to patients), effectiveness (delivering appropriate services), patient-centered care (respecting patients’ values and needs), timeliness (reducing delays and waiting times), efficiency (minimizing waste), and equity (ensuring fair access to care) [18]. Together, these domains complement the SPO model and enable a deeper exploration of the PE, particularly through the processes in which patients interact with healthcare providers (Figure 1).

### 1.3. Healthcare Challenges Faced by DMD Patients

In Mexico, the health system is structured into three main sectors: (1) social security assistance for employed individuals (such as the Mexican Social Security Institute (IMSS) and the Institute of Social Security and Services for State Workers (ISSSTE)); (2) public assistance for those without formal employment, unemployed, or marginalized individuals (including university and local hospitals; specialized national health institutes; and non-profit organizations, such as Teleton, among others); and (3) private healthcare services (hospitals and clinics affiliated with individual-paid insurance plans) [19]. Access to healthcare and the quality of medical services received by families with DMD vary considerably between healthcare systems in different countries. In the United States and the European Union, there are accredited centers for the treatment of DMD. However, in Mexico, only the National Institute of Rehabilitation in Mexico City, a public government institution, has this accreditation. Therefore, non-profit organizations usually cover the needs of those who do not reside in Mexico City [7].

The challenges in delivering healthcare to individuals with DMD in Mexico stem from the condition’s low prevalence, which necessitates molecular laboratory testing and the involvement of various medical specialists, such as pediatricians, neurologists, rehabilitation experts, and geneticists, that in some cases are scarce. For instance, all the states, with the exception of Mexico City, have fewer than one geneticist per 100,000 individuals, falling short of the recommended standard [20].

While international clinical practice guidelines exist to lead the diagnosis, treatment, and management of DMD [1,2,3], the Mexican health system relies predominantly on locally developed guidelines issued by government institutions like the National Center for Technological Excellence in Health (CENETEC). However, despite having guidelines for other genetic diseases, like Pompe disease, among others, CENETEC has not developed specific guidelines for managing DMD in Mexico [21]. Consequently, families affected by DMD in Mexico often contend with a lack of awareness about the condition, absence of nationally endorsed clinical guidelines, and limited access to knowledgeable physicians who can provide appropriate care [7,22].

This study aims to explore both the perceptions of HQ and the PE within Mexico’s DMD community, using Donabedian’s model and the six domains of healthcare quality to understand how structural, process, and outcome factors influence the PE. This approach provided valuable insights into the perceived quality of healthcare services from the unique perspective of individuals living with DMD.

## 2. Materials and Methods

### 2.1. Study Design and Setting

This study used a cross-sectional exploratory research design. Data were collected from 7 to 30 September 2023, through direct invitations to Facebook-based family support groups and non-profit organizations: Enlace Distrofia Muscular Duchenne Becker AC, Sociedad Mexicana para la Distrofia Muscular, and Asociacion de Distrofia Muscular de Occidente A.C. Patients and caregivers were both invited to participate in the study. The inclusion criteria were as follows:

1. Being a patient diagnosed with Duchenne muscular dystrophy or their caregiver.
2. Being over 15 years of age.
3. Being able to speak and read Spanish fluently, as the questionnaire was designed and administered in Spanish.

Participants were excluded if they met the following criteria:

Had uncompensated sensory deficits that prevented them from answering the online questionnaire without help.

Considering that healthcare could come from multiple providers and that this would be challenging for participants when answering the questionnaire, we specifically instructed participants to focus their responses on their current main healthcare provider.

This study was registered with and approved by our institutional research coordination. Participation was voluntary, and the answers from individuals were anonymized for research purposes. The number assigned to this project by local committees was 20-1307-04.

### 2.2. Design of the Instrument

A self-administered questionnaire was developed specifically for this study to assess the perceptions of HQ and PE among DMD patients and caregivers.

A comprehensive literature review was conducted to identify existing instruments that measure patient experience and perceptions of healthcare quality. The search included terms such as “patient experience questionnaire”, “healthcare quality measurement”, and “instruments to measure healthcare quality”. The search was further refined to focus on instruments available in Spanish. One of the instruments was the modified IEXPAC for rare diseases [23]. This instrument evaluates patient and caregiver experiences, particularly healthcare interactions and service quality, and is available in Spanish. However, this questionnaire did not fully address our specific objectives. To address this gap, we reviewed a study by Cunha Ferreira et al. that highlighted key factors influencing patient satisfaction, including the quality of medical care, communication, waiting time, and education [24]. Although patient satisfaction, healthcare quality, and patient experiences are distinct concepts, several dimensions from this review were considered in the design of our novel questionnaire.

An initial version of the questionnaire, comprising 15 items, was developed based on the Donabedian model of quality (structure, process, and outcome) and the STEEEP framework (Safe, Timely, Effective, Efficient, Equitable, and Patient-Centered). To complement the quantitative data, an open-ended question allowed for qualitative exploration of patient experiences. This preliminary version underwent thorough review by researchers, healthcare professionals, and members of the DMD community. Feedback from these stakeholders helped refine the questionnaire, removing redundant and confusing items to ensure clarity and relevance.

### 2.3. Psychometric Properties of the Instrument

The instrument consisted of 10 items measured on a five-point Likert scale. The questionnaire was designed to capture perceptions of healthcare quality across the above-mentioned domains and models. It also included an open-ended question for participants to provide additional qualitative feedback about their experiences with the care received. The items were initially drafted in Spanish, reviewed by the authors, rewritten for clarity, and adapted to ensure cultural appropriateness for Mexican participants. Discrepancies were resolved through team discussion and consensus.

A Bayesian approach was employed to assess the scale’s reliability due to the reduced sample size, calculating McDonald’s Omega and Cronbach’s Alpha that resulted in a final value of 0.87. The analyses were performed using JASP software vesion 0.18.3 [24]. Subsequently, an exploratory factor analysis (EFA) was conducted using Parallel Analysis with Principal Axis Factoring extraction and Oblimin rotation. The EFA revealed a two-factor solution. Factor 1, labeled “Perceived Quality of Healthcare”, encompassed variables such as satisfaction with outcomes, healthcare professional preparedness, and resource utilization efficiency among others. Factor 2, termed “Patient Experience”, included variables related to waiting times, error occurrences, and diagnostic opportunity (Table 1).

The results of the EFA were further validated through a confirmatory factor analysis (CFA), which demonstrated a good fit of the two-factor model to the data. All items exhibited significant factor loadings, supporting the validity of the identified factors. All the analyses were performed using JASP software (Supplementary Materials).

### 2.4. Data Analysis

Descriptive statistics were used to summarize the responses to the questionnaire, using SPSS software (version 28.0). A comparison was conducted between two distinct groups: individuals that receive care services from non-profit organizations and those who were affiliated with a government institution. Participants who were not affiliated with either type of institution or who received care through private hospitals or clinics were excluded from the final comparison due to the low prevalence of individuals in this category (n < 6).

Likert-scale responses were recoded to a 1–5 scale. Subsequently, factor scores were calculated by summing the responses to items within each factor, considering that the item regarding medical errors should be reverse-scored. Exploratory data analysis was conducted to assess data distribution. Finally, the Mann–Whitney U test was employed to compare the total scores of each factor between non-profit and government organizations.

### 2.5. Open-Ended Question Review

Participants were asked an open-ended question to describe their experiences with the healthcare processes for DMD (diagnosis, treatment, follow-up, etc.). Their responses were categorized as positive, negative, or neutral. An additional analysis was then conducted to categorize the responses into the Six Domains of Quality Healthcare. This process involved three steps: (1) initial coding and categorization of individual responses into positive, negative, or neutral by two independent reviewers; (2) categorization of the responses content into the into the Six Domains of Quality Healthcare; and (3) graphical representation of the analysis. Discrepancies in coding or classification were resolved through discussion and consensus among the research team.

## 3. Results

### 3.1. Descriptive Data of the Participants

A total of 91 responses were received, consisting of both DMD patients and caregivers (Table 2). Participants were primarily adults aged 31–55, serving as caregivers for DMD patients, and mostly females. Regarding the institutions that provide medical care, non-profit organizations were preeminent, and the participants were active users of healthcare services. Of the 32 states of Mexico, we received responses from 10. Jalisco, Chihuahua, and Mexico City had the most participants.

### 3.2. Perceived-Quality-of-Healthcare and Patient-Experience Factor Scores Across Institutions

**Figure 2:**
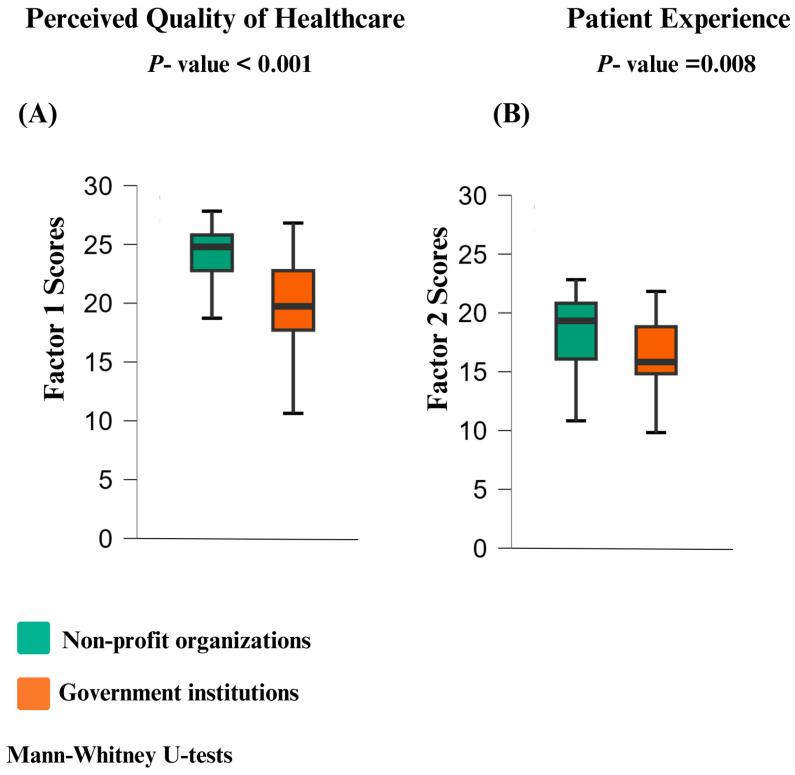
Group comparison of the factors explored by the questionnaire. Green represents non-profit organizations, whereas orange represents government institutions. (**A**) Factor 1 of the questionnaire and (**B**) the second factor of the questionnaire.

The exploratory analysis of the data shows a non-normal distribution. Thus, the Mann–Whitney U test was used to compare total scores for each of the two factors of the questionnaire (perceived quality of healthcare and patient experience) between government institutions and non-profit organizations, the analysis revealed that non-profit organizations scored significantly higher in both factors (Figure 2).

### 3.3. Analysis of the Open-Ended Responses

**Figure 3:**
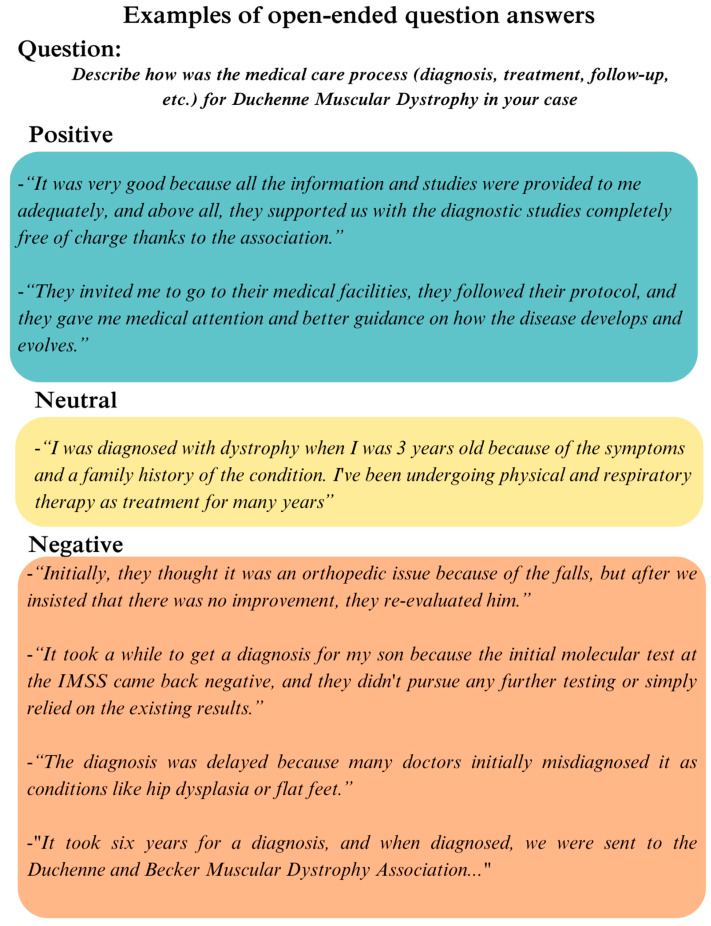
Examples of open responses representative of the dataset. The colors indicate the interpretation used for classification: green for positive, yellow for neutral, and orange for negative.

**Figure 4:**
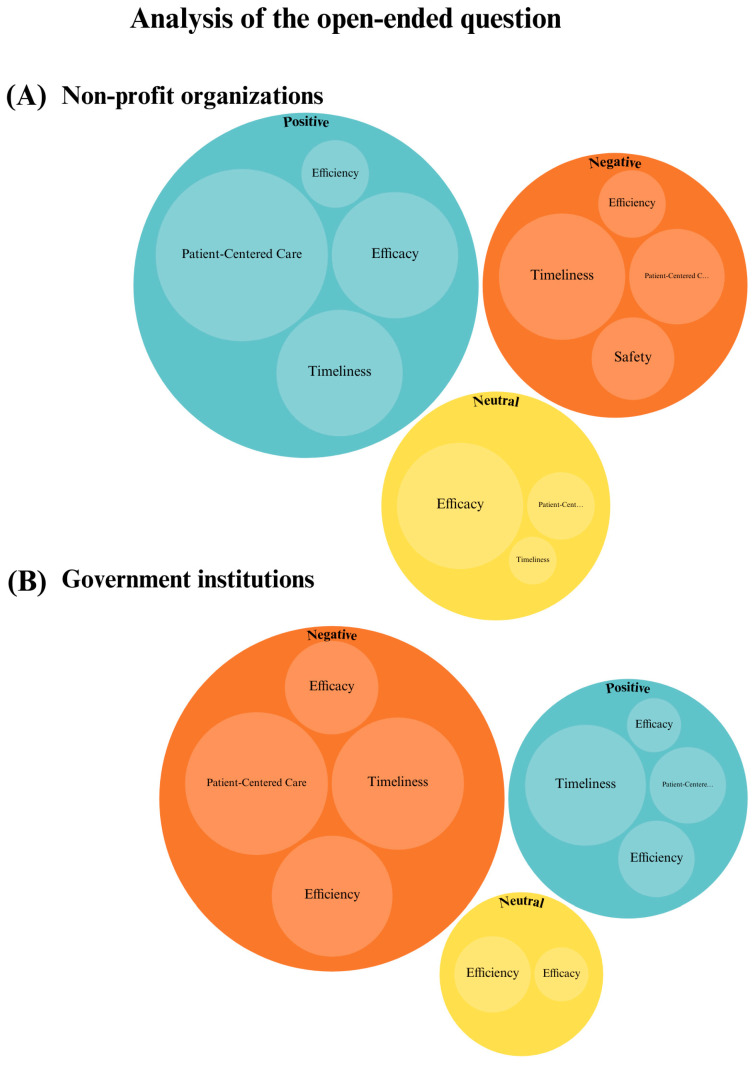
Graphical representation of the open-ended questions analysis. Comparison of responses by institution. The themes were classified as domains of the HQ. Green = positive, yellow = neutral, and orange = negative.

The open-ended responses were classified based on the overall rating of the experience as positive, neutral, or negative (Figure 3) and subsequently associated with elements of the SPO model. For example, “deficient preparation of physicians” was considered a negative experience and linked to the “structure” element, while “rapid diagnosis” was seen as a positive experience and linked to the “process” element. According to the SPO model, the “structure” element includes everything needed to provide medical care, like the preparation and qualifications of physicians. The “process” element includes activities like diagnosis, where health professionals interact with patients. Afterward, a visual representation was created featuring two main sets of circles, one for each group. Within each main circle, smaller circles were used to illustrate the specific SPO elements that best captured the sentiments expressed in the responses (Figure 4).

## 4. Discussion

Patient experience is shaped by various interactions with healthcare providers during diagnosis, treatment, and prevention. Factors such as infrastructure and healthcare professionals’ preparedness significantly influence this experience and patients’ perceptions of HQ. PE affects loyalty to healthcare providers, health outcomes through better treatment adherence, and well-being by meeting patient expectations [11,12,13]. However, measuring patient experience and healthcare quality is challenging due to their reliance on patients’ memories and the personal significance of events.

Many existing questionnaires aim to measure patient experiences (PEs), but they often have limitations, such as being extensive; specific to hospitalized patients; or focused on particular healthcare areas or stages, like diagnosis [17,25,26,27,28]. A qualitative study of DMD adult patients showed how the PE was, regarding how they came to know about their disease and how communication process would improve for better outcomes; nevertheless, no other domains of HQ were explored [9]. In addition, another qualitative study was performed, focused on DMD PE, regarding the loss of physical strength that was the primary defining feature of the disease across all stages of ambulatory ability, but again, no other domains were examined [29]. In addition to the above-mentioned studies, another study was conducted using focus groups. The data suggested that in relation to DMD HQ, there is a need for a team of health professionals (doctors, nurses, physiotherapists, psychologists, etc.) to work together seamlessly within the same unit to provide comprehensive care. This ensures that all aspects of the adolescent’s health are addressed holistically [30]. Nonetheless, a gap in PE perceptions remained.

To improve the quality of medical care for patients with rare diseases, particularly Duchenne muscular dystrophy, it is essential to understand their experiences through easy-to-administer tools that reflect the most relevant aspects to modify during medical care. Herein, we developed a novel questionnaire tailored to the specific experiences of DMD patients, using the Donabedian model and the six domains of healthcare quality [16,18]. This study introduces this innovative questionnaire designed to assess patient perceptions of HQ and PE. The instrument combines quantitative measures using a Likert scale with qualitative data from an open-ended question, providing a deeper understanding of individual experiences and impressions, and it showed appropriate psychometric properties.

The instrument presented here showed that among the Six Domains of Quality, equity and efficiency were classified under Factor 1, related to HQ, while the domains of timeliness, patient-centered care, and safety were classified under Factor 2, related to PE (Table 1 and Figure 2). On the other hand, the study compared the perceptions of two distinct groups: patients receiving care primarily from government institutions and those receiving care primarily from non-profit organizations. Both the factor distribution and the analysis of open-ended responses align because they consistently demonstrated more favorable perceptions of HQ and PE among patients associated with non-profit organizations, as evidenced by both the quantitative Likert-scale responses and the qualitative data that mentioned incidents of perceived medical errors and iatrogenesis that participants remembered as negative experiences. Notably, patient-centered care emerged as a differentiating factor in the PE. Analysis of open-ended responses revealed that patients from non-profit organizations considered this domain positively, while patients from government institutions viewed it as a negative factor (Table 2 and Figure 4).

Interestingly, when reviewing qualitative data, we identified a paradox in patients’ perceptions of timeliness and efficiency because responses revealed that most patients had waited at least two years for an accurate diagnosis, and others had undergone unnecessary tests or received incorrect diagnoses, primarily at government health institutions, but due to the empathy shown by health professionals, the overall sense of the response was positive. This discrepancy suggests that patients may have unclear expectations and tend to accept the care provided despite experiencing delays and inefficiencies. We argue that patient-centered care plays a key role in the positive perceptions of medical care. Both questionnaire data and open-ended responses consistently showed that patients were grateful for the consideration shown by healthcare providers, even though they may not have received optimal HQ. This finding underscores the need for improved patient education and healthcare evaluation and also shows that patient satisfaction does not always reflect the quality of the healthcare. Thus, patient education must improve so that patients can aspire to optimal care standards for this disease, showing in this way that quality domains do not carry the same weight across all cultures and social contexts. In this case, patient-centered care and the human touch provided by healthcare staff can counteract deficiencies in timely diagnosis and other domains.

Our data also suggest that the preparation of health professionals needs to improve specifically in the government institutions. Because among the responses regarding delayed diagnosis and despite the recognition of the DMD as a rare disease in Mexico in May 2023, the absence of national clinical guidelines in Spanish, supported by the Centro Nacional de Excelencia Tecnológica en Salud (CENETEC), poses a challenge to ensuring quality and evidence-based care, mainly for government institutions [31]. Patient associations (non-profit organizations) have taken a leading role by establishing international collaborations with the World Duchenne Organization and Parent Project Muscular Dystrophy and disseminating the most up-to-date standards of care [32]. These efforts may have contributed to the perceived better healthcare reported herein. Therefore, it would be highly beneficial for Mexico to have nation-wide clinical practice guidelines to counteract the perceived deficiencies in the care provided by government institutions for this vulnerable group.

## 5. Limitations of the Study

Efforts were made to distribute the questionnaire as widely as possible among DMD patients and their families through social media support groups and direct outreach via non-profit organizations. However, we acknowledge the potential for sampling bias, as patients and caregivers without access to social media were likely excluded. In addition, a small sample size was used in this study. The instrument was validated only in Spanish; therefore, further studies are required to confirm the usefulness of the presented instrument in other languages.

## 6. Conclusions

Our study offers valuable insights into patients’ perceptions of the quality of their healthcare and introduces a tool to measure these perceptions. Generally, patient perceptions were positive, but notable differences emerged between those affiliated with non-profit organizations and those associated with government institutions. Patients connected with non-profit organizations reported better experiences across several healthcare quality domains. Nonetheless, further research is needed to identify the specific factors contributing to these disparities and to develop strategies to enhance the quality of care across all healthcare settings.

## Figures and Tables

**Table 1:** Questionnaire items and factor loads.

Exploratory Factor Analysis of the Questionnaire Items	Factor Loadings			
Sentence	Target	Perceived Quality of Healthcare (Factor 1)	Patient Experience (Factor 2)	Uniqueness
The preparedness of healthcare professionals to care for patients with DMD is…	Structure	0.863		0.285
The physical condition of the facilities (waiting room, consulting rooms, equipment, areas, offices) for the care of patients with DMD is…	Structure	0.788		0.501
How satisfied are you with the results of the medical care that you or your family member have received?	Outcome	0.697		0.344
I feel that I have received the same level of care for DMD as other people with the same disease, without any distinction based on age, sex, or socioeconomic status.	Process equity	0.613		0.586
Medical care for patients with DMD is characterized by making good use of resources and avoiding waste (laboratory tests, medications, and supplies).	Process efficiency	0.582		0.311
The availability of supplies at the institution that is treating you (reagents, materials, medications, etc.) for the care of patients with DMD is…	Structure	0.420		0.823
Do you consider that the treatment you received for DMD was timely (at the right time for the disease)?	Outcome timeliness		0.921	0.123
Do you consider that the diagnostic process for DMD in your case was timely (the waiting time was reasonable or adequate to know what your or your family member’s disease was)?	Outcome timeliness		0.709	0.554
To what extent have healthcare professionals listened to you and taken your input into account for decisions about DMD medical care?	Process patient-centered care		0.446	0.529
Have you ever experienced any errors on the part of healthcare professionals that have affected the medical care for DMD?	Outcome safety		−0.440	0.690

**Table 2:** Demographic and healthcare-access data.

Variable	Categories	Frequency (%)
Age groups	Younger than 18 years	8 (9.6)
	18–25	7 (8.4)
	26–30	3 (3.6)
	31–35	15 (18)
	36–40	13 (15.6)
	41–55	28 (33.7)
	Older than 56 years	8 (9.6)
	Not specified	1 (1.2)
Sex	Female	68 (81.9)
	Male	15 (18.1)
Participant profile	Patient	13 (15.6)
	Patient relative	70 (84.3)
Health services provider institution	Governmental institution	29 (34.9)
	Non-profit organization	54 (65)
Last health service use	Last week	24 (28.9)
	Last month	32 (38.5)
	6 months ago	8 (9.6)
	More than 6 months ago	8 (9.6)
	More than 2 years ago	11 (13.2)
Average waiting time for scheduling consultations, rehabilitation sessions, or other procedures	One week	35 (42.1)
	Two weeks	8 (9.6)
	One month	14 (16.8)
	Three months	14 (16.8)
	6 months or more	12 (14.4)

## Data Availability

The authors are committed to data sharing and transparency. The dataset underlying the findings of this study is available upon reasonable request from the corresponding author. We encourage researchers to contact us to discuss potential collaborations and data access.

## Supplementary Materials

The following supporting information can be downloaded at https://www.mdpi.com/article/10.3390/healthcare13040412/s1. S1: Instrument Analysis, S2: Questionnaire Responses item by item.
