# Supplementary material for: Perceptions of Healthcare Quality in Duchenne Muscular Dystrophy: A Patient Experience Exploratory Study
Source: Healthcare (Basel). 2025 Feb 14;13(4):412. doi: 10.3390/healthcare13040412 (PMC11855789; doi:10.3390/healthcare13040412)
Supplement: Supplementary file 1 [file healthcare-13-00412-s001.zip › healthcare-3398471-supplementary.pdf]

S1-Instrument Analysis

Bayesian Unidimensional Reliability

Bayesian Scale Reliability Statistics

Estimate	McDonald's ω	Cronbach's α
Posterior mean	0.874	0.873
95% CI lower bound	0.833	0.834
95% CI upper bound	0.910	0.908

Item	McDonald's ω (if item dropped)	Cronbach's α (if item dropped)	Item-rest correlation
	Posterior Mean	Lower 95% CI	Upper 95% CI
The physical condition of the facilities...	866	822	905
The availability of supplies...	875	836	912
The preparation of health professionals...	858	810	899
How satisfied do you feel...?	854	811	897
I feel that I have received the same level of care...	865	820	903
Medical care for DMD patients...	847	799	892
Have you ever experienced any error...?	866	827	908
To what extent have healthcare professionals listened...?	858	816	902
Do you consider that the DMD diagnostic process...?	874	835	911
Do you consider that the treatment you received...?	846	796	890

Exploratory Factor Analysis

Chi-squared Test

	Value	df	p
Model	25.301	26	0.502

Item	Factor 1	Factor 2	Uniqueness
The preparation of health professionals to care for patients with DMD is...	863		285
The physical condition of the facilities...	788		501
How satisfied do you feel...?	697		344
I feel that I have received the same level of care...	613		586
Medical care for DMD patients...	582		311

The availability of supplies...	420		823
Do you consider that the treatment you received...?		921	123
Do you consider that the DMD diagnostic process...?		709	554
To what extent have healthcare professionals listened...?		446	529
Have you ever experienced any error...?		-440	690

Factor Characteristics

		Unrotated solution			Rotated solution		
	**Eigenvalues**	**SumSq. Loadings**	**Proportion var.**	**Cumulative**	**SumSq. Loadings**	**Proportion var.**	**Cumulative**
Factor 1	4.921	4.511	0.451	0.451	3.109	0.311	0.311
Factor 2	1.189	0.743	0.074	0.525	2.145	0.214	0.525

Confirmatory Factor Analysis

Model fit

Chi-square test

**Model**	**X^2**	**df**	**p**
Baseline model	419.217	45	
Factor model	45.195	34	0.095

Note. The estimator is ML.

Parameter estimates

Factor	Indicator	Estimate	Std. Error	z-value	p	Lower 95% CI	Upper 95% CI
Factor 1	The physical condition of the facilities (waiting room, consulting rooms, equipment, areas, offices) for the care of patients with DMD is...	446	71	6.301	<.001	308	585
	How satisfied are you with the results of the medical care that you or your family member have received?	730	79	9.226	<.001	575	885
	I feel that I have received the same level of care for DMD as other people with the same disease, without making	521	79	6.582	<.001	366	676

	distinctions based on age, sex, or socioeconomic level.						
	The availability of supplies in the institution that serves you (reagents, materials, medications, etc.) for the care of patients with DMD is...	344	90	3.812	<.001	167	521
	Medical care for patients with DMD is characterized by making good use of resources and avoiding waste (laboratory tests, medications, and supplies).	755	83	9.154	<.001	594	917
	The preparation of health professionals to care for patients with DMD is...	711	82	8.632	<.001	550	873
Factor 2	Do you consider that the treatment you received for DMD was timely (at the appropriate time for the disease)?	1.136	117	9.707	<.001	907	1.366
	To what extent have healthcare professionals listened to you and taken you into account for decisions about DMD medical care?	650	101	6.413	<.001	451	849
	Have you ever experienced any error by healthcare professionals that affects DMD medical care?	-401	74	- 5.395	<.001	-546	-255
	Do you consider that the DMD diagnostic process in your case was timely (the waiting time reasonable or adequate to know what your or your family member's illness was)?	824	123	6.709	<.001	583	1.065

Factor variances

					95% Confidence Interval	
**Factor**	**Estimate**	**Std. Error**	**z-value**	**p**	**Lower**	**Upper**
Factor 1	1.000	0.000			1.000	1.000
Factor 2	1.000	0.000			1.000	1.000

Factor Covariances

	Estimate	Std. Error	z-value	p	95% Confidence Interval Lower	Upper
Factor 1 - Factor 2	0.754	0.069	10.895	< .001	0.619	0.890

Indicator	Estimate	Std. Error	z-value	p	Lower 95% CI	Upper 95% CI
The physical condition of the facilities (waiting room, consulting rooms, equipment, areas, offices) for the care of patients with DMD is...	293	49	6.015	<.001	198	389
How satisfied are you with the results of the medical care that you or your family member have received?	234	48	4.833	<.001	139	329
I feel that I have received the same level of care for DMD as other people with the same disease, without making distinctions based on age, sex, or socioeconomic level.	360	60	6.005	<.001	242	477
The availability of supplies in the institution that serves you (reagents, materials, medications, etc.) for the care of patients with DMD is...	580	90	6.440	<.001	403	756
Medical care for patients with DMD is characterized by making good use of resources and avoiding waste (laboratory tests, medications, and supplies).	254	54	4.733	<.001	149	359
The preparation of health professionals to care for patients with DMD is...	285	56	5.130	<.001	176	394
Do you consider that the treatment you received for DMD was timely (at the appropriate time for the disease)?	298	130	2.297	22	44	552
To what extent have healthcare professionals listened to you and taken you into account for decisions about DMD medical care?	517	97	5.342	<.001	327	707
Have you ever experienced any error by healthcare professionals that affects DMD medical care?	334	55	6.043	<.001	225	442
Do you consider that the DMD diagnostic process in your case was timely (the waiting time reasonable or adequate to know what your or your family member's illness was)?	810	142	5.703	<.001	532	1.089

Model Fit Measures

RMSEA	RMSEA 90% CI Lower	Upper	TLI	BIC	Model Test χ^2	df	p
0.00	0.00	0.0685	1.01	-121	31.0	34	0.617

S2 Questionnaire Responses item by item

Question	Rating (1-5)	Non-profit Organization	Government Institution
Q1. Physical condition of facilities for DMD care	1	0 (0%)	0 (0%)
	2	0 (0%)	1 (3%)
	3	5 (9%)	9 (31%)
	4	24 (44%)	18 (62%)
	5	25 (46%)	1 (3%)
Q2. Availability of supplies for DMD care	1	0 (0%)	0 (0%)
	2	9 (17%)	6 (21%)
	3	15 (28%)	12 (41%)
	4	26 (48%)	10 (34%)
	5	4 (7%)	1 (3%)
Q3. Preparation of health professionals for DMD care	1	1 (2%)	0 (0%)
	2	1 (2%)	1 (3%)
	3	3 (6%)	15 (52%)
	4	19 (35%)	9 (31%)
	5	30 (56%)	4 (14%)
Q4. Satisfaction with medical care results	1	0 (0%)	0 (0%)
	2	1 (2%)	6 (21%)
	3	5 (9%)	10 (34%)
	4	27 (50%)	11 (38%)
	5	21 (39%)	2 (7%)
Q5. Equity in DMD care	1	0 (0%)	1 (3%)
	2	1 (2%)	0 (0%)
	3	5 (9%)	5 (17%)
	4	18 (33%)	17 (59%)
	5	30 (56%)	6 (21%)
Q6. Efficiency in DMD care	1	0 (0%)	1 (3%)
	2	2 (4%)	2 (7%)
	3	5 (9%)	7 (24%)
	4	17 (31%)	13 (45%)
	5	30 (56%)	6 (21%)
Q7. Safety in DMD care	1	35 (65%)	9 (31%)
	2	9 (17%)	11 (38%)
	3	7 (13%)	6 (21%)

	4	1 (2%)	2 (7%)
	5	2 (4%)	1 (3%)
Q8. Patient-centered care	1	1 (2%)	0 (0%)
	2	4 (7%)	5 (17%)
	3	8 (15%)	7 (24%)
	4	22 (41%)	13 (45%)
	5	19 (35%)	4 (14%)
Q9. Timeliness of diagnosis process	1	4 (7%)	1 (3%)
	2	7 (13%)	7 (24%)
	3	12 (22%)	7 (24%)
	4	12 (22%)	11 (38%)
	5	19 (35%)	3 (10%)
Q10. Timeliness of DMD treatment	1	2 (4%)	2 (7%)
	2	6 (11%)	5 (17%)
	3	7 (13%)	6 (21%)
	4	18 (33%)	12 (41%)
	5	21 (39%)	4 (14%)
